# ‘Trophic’ and ‘source’ amino acids in trophic estimation: a likely metabolic explanation

**DOI:** 10.1007/s00442-017-3881-9

**Published:** 2017-06-06

**Authors:** T. C. O’Connell

**Affiliations:** Department of Archaeology and Anthropology, University of Cambridge, Downing Street, Cambridge, CB2 3DZ UK

**Keywords:** Nitrogen isotopic analysis, Isotope, Metabolism, Trophic discrimination factor, Trophic enrichment factor, Food web

## Abstract

Amino acid nitrogen isotopic analysis is a relatively new method for estimating trophic position. It uses the isotopic difference between an individual’s ‘trophic’ and ‘source’ amino acids to determine its trophic position. So far, there is no accepted explanation for the mechanism by which the isotopic signals in ‘trophic’ and ‘source’ amino acids arise. Yet without a metabolic understanding, the utility of nitrogen isotopic analyses as a method for probing trophic relations, at either bulk tissue or amino acid level, is limited. I draw on isotopic tracer studies of protein metabolism, together with a consideration of amino acid metabolic pathways, to suggest that the ‘trophic’/‘source’ groupings have a fundamental metabolic origin, to do with the cycling of amino-nitrogen between amino acids. ‘Trophic’ amino acids are those whose amino-nitrogens are interchangeable, part of a metabolic amino-nitrogen pool, and ‘source’ amino acids are those whose amino-nitrogens are not interchangeable with the metabolic pool. Nitrogen isotopic values of ‘trophic’ amino acids will reflect an averaged isotopic signal of all such dietary amino acids, offset by the integrated effect of isotopic fractionation from nitrogen cycling, and modulated by metabolic and physiological effects. Isotopic values of ‘source’ amino acids will be more closely linked to those of equivalent dietary amino acids, but also modulated by metabolism and physiology. The complexity of nitrogen cycling suggests that a single identifiable value for ‘trophic discrimination factors’ is unlikely to exist. Greater consideration of physiology and metabolism should help in better understanding observed patterns in nitrogen isotopic values.

## Introduction

Ecologists are increasingly using compound-specific amino acid nitrogen isotopic values as a method for estimating trophic position (Nielsen et al. 2015). The technique relies on the premise of comparing at the intra-individual level the nitrogen isotopic values from amino acids whose δ^15^N values increase with trophic level, and those where the δ^15^N values remain relatively unchanged, the so-called ‘trophic’ and ‘source’ amino acids (Popp et al. 2007; Chikaraishi et al. 2014). In contrast to bulk nitrogen isotopic analysis (Boecklen et al. 2011), this technique produces an internally referenced isotopic measure of trophic position that is argued to have greater precision and accuracy, since it accounts for basal nitrogen isotopic variability in the food web and obviates the need to compare consumer isotopic values to those of potential prey.

The technique has been used to identify dietary intake and trophic position in mammals, birds, insects, reptiles, fish and invertebrates (e.g., Popp et al. 2007; Lorrain et al. 2009; Styring et al. 2010; Chikaraishi et al. 2011; Seminoff et al. 2012; Naito et al. 2013a, b; Steffan et al. 2013; McMahon et al. 2015; Nielsen et al. 2015; Schwartz-Narbonne et al. 2015; McMahon and McCarthy 2016). The majority of studies have used the difference between two amino acids, glutamate and phenylalanine (Glu and Phe), as the two ‘canonical’ ‘trophic’ and ‘source’ amino acids, but some have suggested using a different combination, or a multiple amino acid approach (Popp et al. 2007; Nielsen et al. 2015; McMahon and McCarthy 2016).

**Fig. 1:**
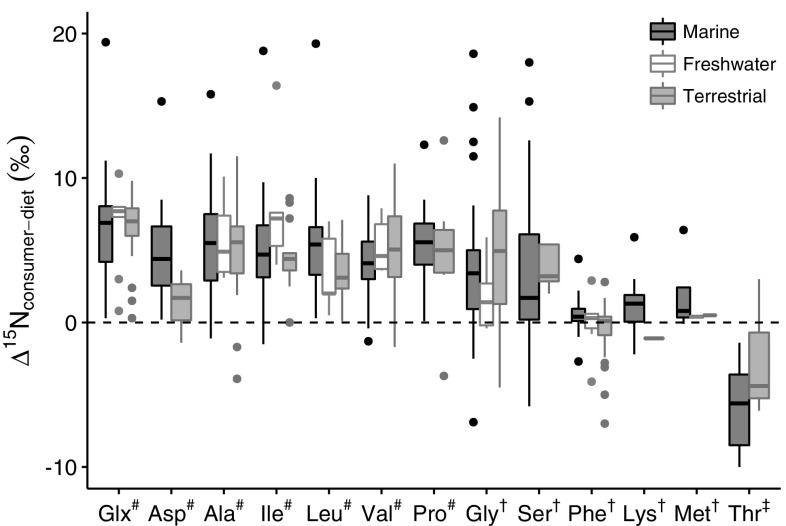
The nitrogen isotopic difference between dietary and body amino acids in controlled feeding studies and well-constrained field collections. Data from McMahon and McCarthy (2016) are shown as boxplots for each amino acid, plotted by ecosystem type (marine, freshwater, terrestrial), where the* shaded box* represents the inter-quartile range (IQR), the whiskers represent the values within 1.5× IQR of the upper or lower quartiles, and outlier values beyond the end of the whiskers are plotted as points. ‘Trophic’ amino acids are marked with a hash, ‘source’ amino acids are marked with a dagger, and the ‘metabolic’ amino acid is marked with a double dagger. *Glx* glutamate, *Asp* aspartic acid, *Ala* alanine, *Ile* isoleucine, *Leu* leucine, *Val* valine, *Pro* proline, *Gly* glycine, *Ser* serine, *Phe* phenylalanine, *Lys* lysine, *Met* methionine, *Thr* threonine

In using new methodologies, it is imperative to ensure that the technique is built on firm foundations. Whilst the general concept behind amino acid nitrogen isotopic analysis seems to be robust, based on the studies published so far, there are some hints that nitrogen isotopic patterning in amino acids is not as simple or consistent as initially suggested. For instance, in early discussions of the method, it was suggested that there is minimal difference in the nitrogen isotopic values of dietary Phe and Phe in body tissues (Chikaraishi et al. 2009, 2014), yet controlled studies have shown that Phe in consumers can have significantly lower nitrogen isotopic values than dietary Phe, although the Glu–Phe offset can be consistent (Steffan et al. 2013). A recent review of published amino acid nitrogen isotopic analyses from controlled studies shows that nitrogen isotopic difference between amino acids in the diet and the consumer can vary considerably, for both ‘trophic’ and ‘source’ amino acids, correlated with factors such as trophic position, habitat, dietary quality and nitrogen excretion mechanism (Fig. 1; McMahon and McCarthy 2016).

As illustrated in the “Proxy Confidence Factor Phase Chart” proposed by the eminent geochemist Harry Elderfield, confidence in any proxy moves through a series of phases (from optimism to pessimism and then to realism) as the relevant research develops (Elderfield 2002). In order to use any new proxy to its full potential, we need to move into the ‘realism phase’ as fast as possible, and I argue that a greater understanding of the metabolic underpinnings of amino acid nitrogen isotopic analysis is critical to its development as a useful method.

But understanding the patterning in nitrogen isotopic values of amino acids is not just relevant to such compound-specific work but also to that of bulk tissue isotopic analyses, since measured bulk protein nitrogen isotopic values are weighted averages of individual amino acid nitrogen isotopic values in the particular tissue analysed. Ecological and ecophysiological studies using nitrogen isotopic data have addressed such questions as foodweb structure, trophic position, dietary composition, resource use and allocation, seasonal and ontogenic variation in resource utilisation, nutritional adequacy and restriction, as well as specialisation (Dalerum and Angerbjorn 2005; Newsome et al. 2007; Araujo et al. 2011; Boecklen et al. 2011; Hammerschlag-Peyer et al. 2011; Layman et al. 2012, 2015; Hertz et al. 2015; Thomas and Crowther 2015; Vander Zanden et al. 2015). So beyond the question of confidence in a new proxy, elucidating the metabolic drivers of isotopic patterning will allow greater insight into how observed nitrogen isotopic signals emerge from the myriad biochemical reactions that occur between food consumption and tissue synthesis. As Martínez del Rio and Anderson-Sprecher suggested almost a decade ago, “Progress in the study of isotopic incorporation demands that we understand the process of incorporation mechanistically. We find the significance of isotopic incorporation information in field ecological studies. We will find its meaning in physiological research” (Martínez del Rio and Anderson-Sprecher 2008). Thus, a better understanding of nitrogen metabolism is highly pertinent for all nitrogen isotopic studies in ecology and eco-physiology, whether the work is carried out at a bulk tissue or at an amino acid level.

## The ‘trophic’ and ‘source’ isotopic groupings of amino acids

So far the literature contains suggestions for the reasons for the observed nitrogen isotopic patterning in amino acids, but no clear consensus as to the underlying metabolic mechanism. The ‘trophic’ amino acids are usually taken to be glutamic acid, alanine, aspartic acid, proline, leucine and valine and the ‘source’ amino acids to be phenylalanine, glycine, serine, tyrosine, lysine, methionine and histidine (Popp et al. 2007; Nielsen et al. 2015). Threonine has been categorised as a ‘source’ amino acid (Nielsen et al. 2015) but it has long observed to have anomalous nitrogen isotopic values, often extremely low in comparison to other amino acids within the same protein (Gaebler et al. 1966; Tuross et al. 1988; Hare et al. 1991; O’Connell and Hedges 2001) and has more recently been described as a ‘metabolic’ amino acid because it shows substantial nitrogen isotopic depletion relative to dietary threonine (Germain et al. 2013; McMahon et al. 2015). Here, I shall use the term ‘metabolic’ to describe threonine, even though I consider it something of a misnomer, since all amino acids are ‘metabolic’ in that they are involved in metabolic processes.

It is clear that the two groups of ‘trophic’ and ‘source’ (or even a comparison of ‘trophic’ vs. ‘source’ and ‘metabolic’) do not map neatly onto many other categorizations of amino acids, including that of non-essential/essential (also known as dispensable/indispensable), or of glucogenicity/ketogenicity, hydrophilicity/hydrophobicity, polarity or stereochemistry (e.g. branched chain/aliphatic). For any grouping that pertains to nitrogen isotopic values, it is unlikely that the underlying mechanism relates to any chemical or biochemical categorization based on carbon structures, since primary nitrogen isotope effects can only occur when bonds involving nitrogen are broken and formed.

The most plausible reason for nitrogen isotopic variation between amino acids is different degrees of isotopic fractionation associated with nitrogen transfer, in particular transamination or deamination, as has been conjectured widely across the literature (Gaebler et al. 1966; Macko et al. 1986, 1987; Hare et al. 1991; Metges and Petzke 1997; Schoeller 1999; O’Connell and Hedges 2001; McClelland and Montoya 2002; Petzke et al. 2005; Chikaraishi et al. 2007; Styring et al. 2010; Braun et al. 2014; Poupin et al. 2014; McMahon and McCarthy 2016). Kinetic isotope effects for nitrogen at natural abundance have been demonstrated in vitro for reactions catalysed by aspartate transaminase (glutamic oxaloacetic transaminase) and glutamine synthetase (Macko et al. 1986; Yoneyama et al. 1993).

The ‘trophic’/‘source’ dichotomy has been suggested to indicate the split between those amino acids that undergo transamination and those that do not (Chikaraishi et al. 2007, 2009; Braun et al. 2014). However, the ‘trophic’/‘source’ groupings also do not map directly onto the transaminating and deaminating amino acids, particularly as regards proline and phenylalanine (see later discussion).

Here I argue that it is not just the ability of an amino acid to be transaminated that is key to the isotopic patterning that we see, rather the degree to which the amino-nitrogen in each amino acid can be ‘cycled’ through the system. As was pointed out over three decades ago, transamination reactions for virtually all amino acids have been identified in vitro, but it does not follow that such a mechanism is functionally significant for each amino acid in vivo (Jackson 1983), something that was overlooked in a recent paper on the metabolism underlying amino acid nitrogen isotopic variability (Braun et al. 2014). Drawing on the concept of the metabolic pool of nitrogen, first proposed by Sprinson and Rittenberg (1949), I postulate that ‘trophic’ amino acids are those whose amino-nitrogen can be considered to be interchangeable, part of a metabolic amino-nitrogen pool, and that ‘source’ amino acids are those whose amino-nitrogens are not interchangeable with the metabolic pool.

## Amino acid nitrogen metabolism

### ‘Trophic’ amino acids

Those amino acids whose α-nitrogens are interchangeable are primarily linked via glutamic acid. Glutamic acid is central to nitrogen metabolism, being the route by which amino acid nitrogen enters the urea cycle (Brosnan 2000); it is key in the transamination of many amino acids (Cammarata and Cohen 1950), with the reaction catalysed by glutamate dehydrogenase (Hudson and Daniel 1993). Glutamine is rarely mentioned as a ‘trophic’ amino acid, yet is formed from the addition of ammonia to glutamic acid (Bertolo and Burrin 2008): glutamine’s α-nitrogen (one of its two nitrogen atoms) is therefore de facto in the ‘trophic’ category, something that is often overlooked given that the measured glutamate of any sample analysed after acid hydrolysis is derived from both glutamic acid and deamidated glutamine (thus strictly speaking is ‘Glx’), Four of the ‘trophic’ amino acids, alanine, aspartate, leucine and valine (as well as isoleucine), readily transaminate with α-keto-glutarate, forming their respective keto-acids and glutamic acid (Cammarata and Cohen 1950; Harper and Zapalowski 1981). Proline cannot transaminate because its secondary amino group is part of a ring structure, but its amino-nitrogen (and that of its post-translationally modified variant hydroxyproline: Gorres and Raines 2010; O’Connell and Collins 2017) is derived from the same pool as that of glutamic acid α-nitrogen, since proline is synthesised via ring closure from glutamic acid, and the reaction is reversible (Bertolo and Burrin 2008).

### ‘Source’ and ‘metabolic’ amino acids

The ‘source’ amino acids, as well as the ‘metabolic’ threonine, do not typically participate in α-amino transamination in vivo, and thus the only mechanism by which amino-nitrogen can be removed is through catabolism. Glycine readily interconverts with serine via the enzyme serine hydroxymethyl transferase and may be derived from threonine via the action of the enzyme threonine dehydrogenase (Neuberger 1961). These three amino acids are normally catabolized by irreversible deamination through several possible routes, the glycine-cleavage system, or via serine dehydratase, serine-pyruvate aminotransferase or threonine dehydratase (Walsh and Sallach 1966; Kikuchi 1973; Bird and Nunn 1983; Snell 1986). Histidine is catabolized by deamination by histidine ammonia lyase to produce ammonia and urocanate, which subsequently forms glutamate, such that histidine’s imidazole-nitrogen becomes the α-amino-nitrogen of glutamate (Mehler and Tabor 1953; Revel and Magasanik 1958; Coote and Hassall 1973). Although methionine transamination of unknown mechanism has been detected in vivo (Blom et al. 1989; Brosnan and Brosnan 2006), methionine is predominantly catabolized via the transmethylation-transsulfuration pathway. In this, methionine is converted to homocysteine, which condenses with serine to produce cystathionine which is enzymatically cleaved to yield cysteine: methionine’s sulphur atom is thus transferred into cysteine, whilst the carbon skeleton becomes α-ketobutyrate and the amino group is released as ammonia (Cooper 1983; Stipanuk 1986, 2004). Thus, for the five amino acids glycine, serine, threonine, histidine and methionine, the amino-nitrogen is converted to ammonia. This ammonia is not necessarily ‘lost’ from the metabolic amino-nitrogen pool, as free ammonia can be recycled via incorporation into glutamine or glutamic acid (Hudson and Daniel 1993; Bertolo and Burrin 2008).

For lysine, which has two amino groups, degradation occurs by irreversible transamination of the ε-amino group with α-keto-glutarate via the intermediate of saccharopine to produce glutamate and α-aminoadipate 6-semialdehyde (allysine), which is then dehydrated to α-amino-adipate, which transaminates with α-keto-glutarate to produce glutamate and α-keto-adipate (Fellows and Lewis 1973; Carson 1974). Thus both nitrogens of lysine transfer to the metabolic nitrogen pool via incorporation in glutamate.

The two remaining ‘source’ amino acids, phenylalanine and tyrosine, are both indispensable and aromatic and are metabolically interlinked. Phenylalanine has two potential catabolic routes, a minor one involving transamination with pyruvate to form phenylpyruvate and alanine, and a dominant one involving hydroxylation to tyrosine, which is irreversible (Krempf et al. 1990; Matthews 2007). Tyrosine aminotransferase catalyses the reaction between tyrosine and α-keto-glutarate to form p-hydroxyphenylpyruvate and glutamate (Matthews 2007) and thus via this route, the α-nitrogens of both phenylalanine and tyrosine are donated to the metabolic nitrogen pool by incorporation in glutamic acid during irreversible catabolism.

## Evidence for in vivo nitrogen exchange between amino acids

**Fig. 2:**
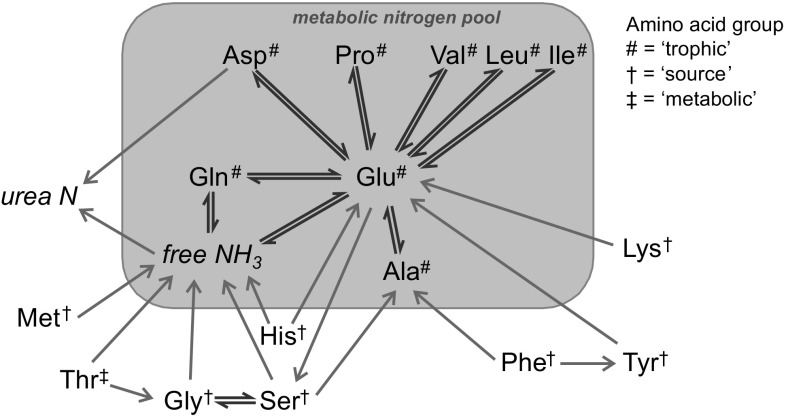
Schematic representation of the metabolic fate of nitrogen in ‘trophic’ and ‘source’ amino acids in mammals. Routes of nitrogen movement between ‘trophic’ and ‘source’ amino acids and other key nitrogen species are shown, with exchange represented by ⇌, and unidirectional movement (e.g. irreversible transamination or deamination) by →. Those amino acids whose nitrogen can be readily exchanged in a single chemical step, the ‘trophic’ amino acids (marked with a hash), are connected to other ‘trophic’ amino acids by ⇌, and together with free ammonia, they form the metabolic amino-nitrogen pool, outlined in the *shaded box*. ‘Source’ amino acids (marked with a dagger), and the ‘metabolic’ amino acid (marked with a double dagger) can also donate nitrogen to other amino acids and to the metabolic pool, but cannot readily *exchange* nitrogen (except between glycine and serine): all such amino acids are outside the metabolic amino-nitrogen pool. *Glu* glutamic acid, *Gln* glutamine, *Asp* aspartic acid, *Ala* alanine, *Ile* isoleucine, *Leu* leucine, *Val* valine, *Pro* proline, *Gly* glycine, *Ser* serine, *Phe* phenylalanine, *Tyr* tyrosine, *Lys* lysine, *Met* methionine, *Thr* threonine, *His* histidine

The overall metabolic picture of amino acid nitrogen is therefore one of exchange or lack of it, a dichotomy that has been proposed before (Jackson and Golden 1980). The transaminating amino acids (alanine, aspartate, leucine, valine and isoleucine), together with proline, exchange nitrogen with glutamic acid, and thus also with glutamine. These amino acids, together with free ammonia, form the metabolic nitrogen pool (Fig. 2). The ‘source’ and ‘metabolic’ amino acids do not freely exchange nitrogen with the other (transaminating) amino acids, but can donate nitrogen in some form to the metabolic pool, either by irreversible transamination with α-keto-glutarate (directly in the case of histidine, lysine and tyrosine, or via an intermediate for phenylalanine) or with pyruvate (for serine), or by the production of ammonia (glycine, serine, threonine, histidine, methionine) (Fig. 2). Serine and glycine can also derive nitrogen from the metabolic pool, as the biosynthesis of serine derives the amino group from glutamate in an irreversible transamination catalysed by phosphoserine amino transferase (Snell 1986).

Evidence for the degree of in vivo nitrogen exchange between amino acids comes from the wealth of information available from isotopic tracer studies of protein metabolism and physiology over the past 80 years (Schoenheimer 1942; Waterlow 2006); such work is no doubt key to understanding the observed natural abundance isotopic signals in ecology, especially at the molecular level. Studies using ^15^N-labelled amino acids allow us to go beyond the identification of which pathways are metabolically possible, and to assess the extent to which nitrogen exchange occurs between amino acids in vivo—the concept of ‘flux’ is critical here, since metabolic maps “tell us ‘what goes’ but not ‘how much’” (Kacser and Burns 1973; Kacser et al. 1995).

Labelling studies show that nitrogen freely exchanges between glutamic acid, proline, alanine, aspartic acid and the branched chain amino acids (leucine, isoleucine, valine) with the patterning of the nitrogen label across amino acids, ammonia and urea suggesting that the exchange is direct, i.e. via transamination (Aqvist 1951b; Matthews et al. 1981a; Darmaun et al. 1986; Cooper et al. 1987, 1988). Studies show that histidine and lysine do not acquire nitrogen from other amino acids or ammonia (Elliott and Neuberger 1950; Aqvist 1951b; Fern et al. 1985), nor does their amino-nitrogen freely pass to other amino acids, rather that any nitrogen that does pass from them to other amino acids is likely to be via free ammonia and subsequent incorporation into the metabolic pool (Meltzer and Sprinson 1952). Similar results are obtained for glycine, serine, threonine, phenylalanine and tyrosine, although with some differences due to catabolic pathways: glycine and serine readily interchange nitrogen, and both can receive nitrogen from threonine as a result of the glycine-cleavage system; tyrosine nitrogen can originate in phenylalanine (Elliott and Neuberger 1950; 1951a, b; Meltzer and Sprinson 1952; Jackson and Golden 1980; Matthews et al. 1981b; Lehmann and Heinrich 1985; Matthews 2007).

Critically, exchange of nitrogen between transaminating amino acids is fast, as are the turnover rates of amino acids in free pools and tissues, of the order of minutes or hours depending on the amino acid and the pool, as well as the species (see Chapters 3 and 4 in Waterlow 2006). Studies using short-lived isotopes suggest that the turnover and equilibration can be even more rapid, of the order of seconds (Cooper et al. 1987, 1988). When labelled [^13^N]glutamate was injected into the portal vein of rats, after 60 s the tracer was already widely distributed across amino acids in the liver: 21% of the tracer was in aspartate, 14% in glutamine and 10% in alanine (Cooper et al. 1988). Thus, nitrogen cycling through the metabolic nitrogen pool is a constant and high-frequency process.

## Heterotrophic nitrogen metabolism

The isotopic evidence I describe here is primarily derived from studies of mammalian protein metabolism, and one has to ask how relevant these are to other heterotrophs. Yet metabolic pathways are highly conserved in nature (Peregrin-Alvarez et al. 2009).

Glutamate, key to trophic position estimates from isotopic data, is a core molecule in metabolic functioning throughout all living organisms, linking protein and energy metabolism (Brosnan 2000): it is central to the transamination of many amino acids, and its keto-acid, α-keto-glutarate, is a key intermediate in the Krebs (citric acid) cycle (Young and Ajami 2000). In studies of metabolic network function based on genomic structural data, glutamate is the most connected metabolite in *Escherichia coli* (Wagner and Fell 2001) and is in the top ten most connected substrates for 41 of 43 organisms studied across the three domains (archaea, bacteria and eukaryotes) (Jeong et al. 2000). This, together with the near universal distribution of glutamate dehydrogenases across the three domains (Hudson and Daniel 1993), indicates glutamate’s centrality as a metabolic intermediate over a long evolutionary timescale (Young and Ajami 2000).

Some key metabolic differences in amino acids no doubt occur (e.g. the occurrence of alanine dehydrogenases in insects and bacteria but not mammals, and lysine metabolism in fungi), but nitrogen isotope studies on amino acids from diverse species including bacteria, fungi, fish and birds echo the patterns seen in the larger mammalian literature using labelled ^15^N compounds (Bent 1964; Richter and Gruhn 1977; Gruhn 1987; Macko et al. 1987; Chalot et al. 1995; Iwata and Deguchi 1995; Rodicio et al. 2003). Thus, although there are metabolic variations across heterotrophs, the evidence suggests that the mechanism outlined here for the patterning in ‘trophic’ and ‘source’ amino acids is probably pertinent across most organisms.

There has been a suggestion that amino acids could be categorised into four groups in terms of their essentiality, following a two-way grouping structure, one that relates to synthesis of the carbon skeleton, and one that relates to amination of the skeleton (Jackson 1983). This fits plausibly with the basis of ‘trophic’ and ‘source’ amino acids proposed here. It also fits with observations that the categorisation of amino acids into ‘trophic’ and ‘source’ is not always clear cut (McMahon and McCarthy 2016), since the essentiality of any given amino acid varies between species and can change with age and illness within a species (National Research Council 1994, 1995; Young and Borgonha 2000).

## What do the isotopic signals represent?

The exchange of nitrogen across transaminating amino acids and proline (and post-translationally modified variants such as hydroxyproline) means that the nitrogen isotopic values of the ‘trophic’ amino acids in a consumer do not simply represent the nitrogen isotopic value of those same amino acids in the diet plus an isotopic offset due to metabolism of that amino acid, as is usually assumed (McClelland and Montoya 2002; Chikaraishi et al. 2007, 2009, 2014; Popp et al. 2007; Steffan et al. 2013, 2015; Vander Zanden et al. 2013; Hoen et al. 2014; Nielsen et al. 2015). Rather, because of the degree of nitrogen cycling through the metabolic nitrogen pool, there is an averaging effect: the nitrogen isotopic values of amino acids that can exchange nitrogen reflect an averaged isotopic signal of all such amino acids in the diet, offset by the integrated effect of isotopic fractionation from multiple transaminations during constant nitrogen cycling. This isotopic signal of any particular amino acid in any particular body tissue or pool will also be modulated by subtle isotopic shifts associated with a number of other factors depending on the individual: physical and metabolic compartmentation within and between tissues (Fern and Garlick 1976; Fern et al. 1985; Hoskin et al. 2001; Poupin et al. 2014); the balance of protein synthesis and oxidative loss of amino acids (Felig 1975; Millward and Rivers 1989; Poupin et al. 2014); potential input of nitrogen from other amino acids via irreversible transamination or incorporation of free ammonia (either directly into glutamine or glutamic acid (Aqvist 1951b), or via microbial synthesis (Torrallardonna et al. 1996; Fuller and Reeds 1998; Metges et al. 1999; Cantalapiedra-Hijar et al. 2016)); the ‘scavenging’ of urea from the gut (Fuller and Reeds 1998); and the mechanisms and route(s) of nitrogen excretion (Schoeller 1999; Vanderklift and Ponsard 2003; Germain et al. 2013; McMahon et al. 2015; McMahon and McCarthy 2016). The centrality of glutamate to heterotrophic nitrogen metabolism is strong evidence as to why it is the key canonical ‘trophic’ amino acid across all organisms analysed so far.

The nitrogen isotopic value of the ‘source’ amino acids should be more closely linked to that of the same amino acid in the diet, without the isotopic effects due to nitrogen cycling, but also subtly modulated by the same shifts affecting the ‘trophic’ amino acids (physical and metabolic compartmentation, protein synthesis vs. oxidation, microbial synthesis), as well as specific effects due to their catabolic pathways, as well as de novo synthesis rates for serine and glycine. The variability in the metabolic processing of amino acids between individuals, classes, species, is a likely explanation for the high degree of variability in ‘source’ amino acids (e.g. Gly nitrogen isotopic values relative to ‘trophic’ amino acids in both terrestrial and aquatic systems, see Fig. 1, also Styring et al. 2010; Nielsen et al. 2015; Steffan et al. 2015; McMahon and McCarthy 2016). Some ‘source’ amino acids appear to have a more faithful isotopic relationship to that of the dietary amino acid (e.g. Phe) than others, which may well relate to the limited number of metabolic pathways in which they are involved (see Fig. 2), but it is possible that there is no one single ‘source’ amino acid that can be considered as the best ‘source’ amino acid for every organism type.

The isotopic signal of threonine, the sole ‘metabolic’ amino acid as currently defined, is highly variable, and as yet, the reason is unknown. Recent work indicates that it is not due to an inverse enzymatic isotope effect, but may result from organismal, rather than cellular, metabolism of threonine, possibly including microbial synthesis (Wallace and Hedges 2016).

The importance of nitrogen cycling in amino acid nitrogen isotopic variation has been suggested before (Schwarcz and Schoeninger 1991; Fogel et al. 1997; McMahon and McCarthy 2016). That the similarity between the nitrogen isotopic values of transaminating amino acids and proline likely reflects the common origin of their amino groups in glutamate has also previously been postulated (Styring et al. 2010; Braun et al. 2014).

The idea of ‘averaging’ of the nitrogen isotopic signal within the metabolic nitrogen pool chimes with studies examining whether trophic position can be better estimated using nitrogen isotopic data from multiple amino acids (Nielsen et al. 2015; McMahon and McCarthy 2016): the use of multiple amino acids will average out some of the biological noise, but it may also better reflect the reality of the nitrogen cycling between amino acids. However, this again emphasises the complexity of the underlying metabolism and thus the complexity of the origin of the isotopic signal that we observe. As Poupin et al.’s work on compartmental modelling of inter-tissue nitrogen fluxes shows, ^15^N accumulation in tissues cannot be sufficiently explained by isotopic fractionation associated with amino acid catabolism and nitrogen elimination within simple whole-body one-compartment models (Poupin et al. 2014). This is as true at the individual amino acid level as at the bulk tissue level.

## Conclusion

Evidence from both natural abundance and labelled isotopic work shows that the ‘trophic’ and ‘source’ amino acid groupings have a fundamental metabolic origin, to do with the metabolic pathways of amino acids, and the cycling of amino-nitrogen through the metabolic nitrogen pool.

Currently, a strong focus within the isotopic ecology community appears to be a drive to better ‘pin down’ the diet-consumer isotopic difference in amino acids across many species, and, therefore, to quantify more precisely the so-called ‘trophic discrimination factors’ for amino acids, possibly even to identify a single value. Multiple studies show clear patterns in intra-individual amino acid nitrogen isotopic values across a wide range of species (Nielsen et al. 2015), often generating a narrow range of values for trophic discrimination factors (Chikaraishi et al. 2014) which are sometimes very consistent and reproducible (Steffan et al. 2013, 2015). However, I argue that, given the complexity of nitrogen cycling through the metabolic network, it is doubtful that a single fixed value for these trophic discrimination factors exists, especially when attempting to extrapolate across all species, families, classes, even phyla. Such discrimination factors are likely to correlate with a variety of potential causes (Nielsen et al. 2015; McMahon and McCarthy 2016), and a quest for a single value may be chasing after a chimera. A greater degree of insight will probably be achieved by exploring the variability in amino acid nitrogen isotopic patterns in conjunction with physiological and metabolic studies.
